# Ion–ion interactions in the denatured state contribute to the stabilization of CutA1 proteins

**DOI:** 10.1038/s41598-018-25825-7

**Published:** 2018-05-16

**Authors:** Katsuhide Yutani, Yoshinori Matsuura, Hisashi Naitow, Yasumasa Joti

**Affiliations:** 1 RIKEN SPring-8 Center, 1-1-1 Kouto, Sayo, Hyogo, 679-5148 Japan; 2 Japan Synchrotron Radiation Research Institute, 1-1-1, Kouto, Sayo, Hyogo, 679-5198 Japan

## Abstract

In order to elucidate features of the denatured state ensembles that exist in equilibrium with the native state under physiological conditions, we performed 1.4-μs molecular dynamics (MD) simulations at 400 K and 450 K using the monomer subunits of three CutA1 mutants from *Escherichia coli*: an SH-free mutant (Ec0SH) with denaturation temperature (*T*_d_) = 85.6 °C, a hydrophobic mutant (Ec0VV) with *T*_d_ = 113.3 °C, and an ionic mutant (Ec0VV_6) with *T*_d_ = 136.8 °C. The occupancy of salt bridges by the six substituted charged residues in Ec0VV_6 was 140.1% at 300 K and 89.5% at 450 K, indicating that even in the denatured state, salt bridge occupancy was high, approximately 60% of that at 300 K. From these results, we can infer that proteins from hyperthermophiles with a high ratio of charged residues are stabilized by a decrease in conformational entropy due to ion–ion interactions in the denatured state. The mechanism must be comparable to the stabilization conferred by disulfide bonds within a protein. This suggests that introduction of charged residues, to promote formation of salt bridges in the denatured state, would be a simple way to rationally design stability-enhanced mutants.

## Introduction

Despite many advances in the field of protein engineering^1,2^, ways to improve the stability of a protein have not been established, perhaps due to a lack of knowledge about the denatured state. The denatured state exists in equilibrium with the native state at physiological conditions, but the conformational structures of a denatured protein are seldom studied because these molecules are such a small fraction of the population, approximately 1/10^8^ molecules when Δ*G* of unfolding is 50 kJ/mol^3–6^.

It is known that the proteins of hyperthermophiles, which grow at temperatures exceeding 80 °C, have a greater proportion of charged residues as compared with those of mesophiles^7,8^. Although this fact has been widely reported^9–17^, how the abundance of charged residues contributes to the stabilization of proteins from hyperthermophiles remains unclear. Disulfide bonds are believed to conformationally stabilize proteins by decreasing the entropy of the denatured state^18^. Hence, we investigated whether ion–ion interactions between charged residues in hyperthermophiles have a similar effect.

The CutA1 protein was originally identified as the product of the *cutA* gene locus of *Escherichia coli*, which is involved in divalent metal tolerance^19^. The specific function of CutA1 in *E*. *coli* is still unknown. CutA1 homologs have been identified in bacteria, plants, and animals, including humans^20^. The CutA1 protein from the hyperthermophile *Pyrococcus horikoshii* (PhCutA1) exhibits unusually high stability, with a denaturation temperature (*T*_d_) of approximately 150 °C at pH 7.0^20^. The percentage of charged residues is 42% of the amino acids in PhCutA1, considerably higher than the value of 25% for CutA1 from *E*. *coli* (EcCutA1)^20^. These characteristics make PhCutA1 a good model protein for investigating the role of charged residues in the stability of proteins from hyperthermophiles^12,21–24^. Comprehensive studies of PhCutA1 mutants have shown that substitution of positively charged residues (Lys, Arg) with uncharged residues results in greater changes in average *T*_d_ (Δ*T*_d_) than substitution of negatively charged residues (Glu, Asp). Indeed, the average Δ*T*_d_ of 21 Glu mutants was negligible, at 0.03 ± 2.05 °C^12^. This suggests that negatively charged residues are forced to be partially repulsive to each other. However, these negatively charged residues, which do not appear to impact *T*_d_, may be important for maintaining the physiologically important isoionic point (p*I*). To examine the stabilization mechanism of this extremely stable protein, EcCutA1 mutants have been designed in order to construct a protein with a denaturation temperature similar to that of PhCutA1^23^.

The unusually high ratio of charged residues in hyperthermophile proteins might contribute to protein stability through an effect on the denatured state ensemble. We performed molecular dynamics (MD) simulation^25–31^ to elucidate the characteristics of charged residues in the denatured state, using three *E*. *coli* CutA1 mutants: an SH-free mutant with *T*_d_ = 85.6 °C (Ec0SH), a hydrophobic mutant also lacking SH groups with *T*_d_ = 113.3 °C (Ec0VV), and an ionic mutant with *T*_d_ = 136.8 °C (Ec0VV_6), as previously reported^23^. The hydrophobic mutant Ec0VV differs from Ec0SH by only two residues and has a *T*_d_ 27.7 °C higher than that of Ec0SH. The ionic mutant Ec0VV_6 differs from Ec0VV by the addition of six charged residues and has a *T*_d_ 23.5 °C higher than that of Ec0VV.

**Figure 1:**
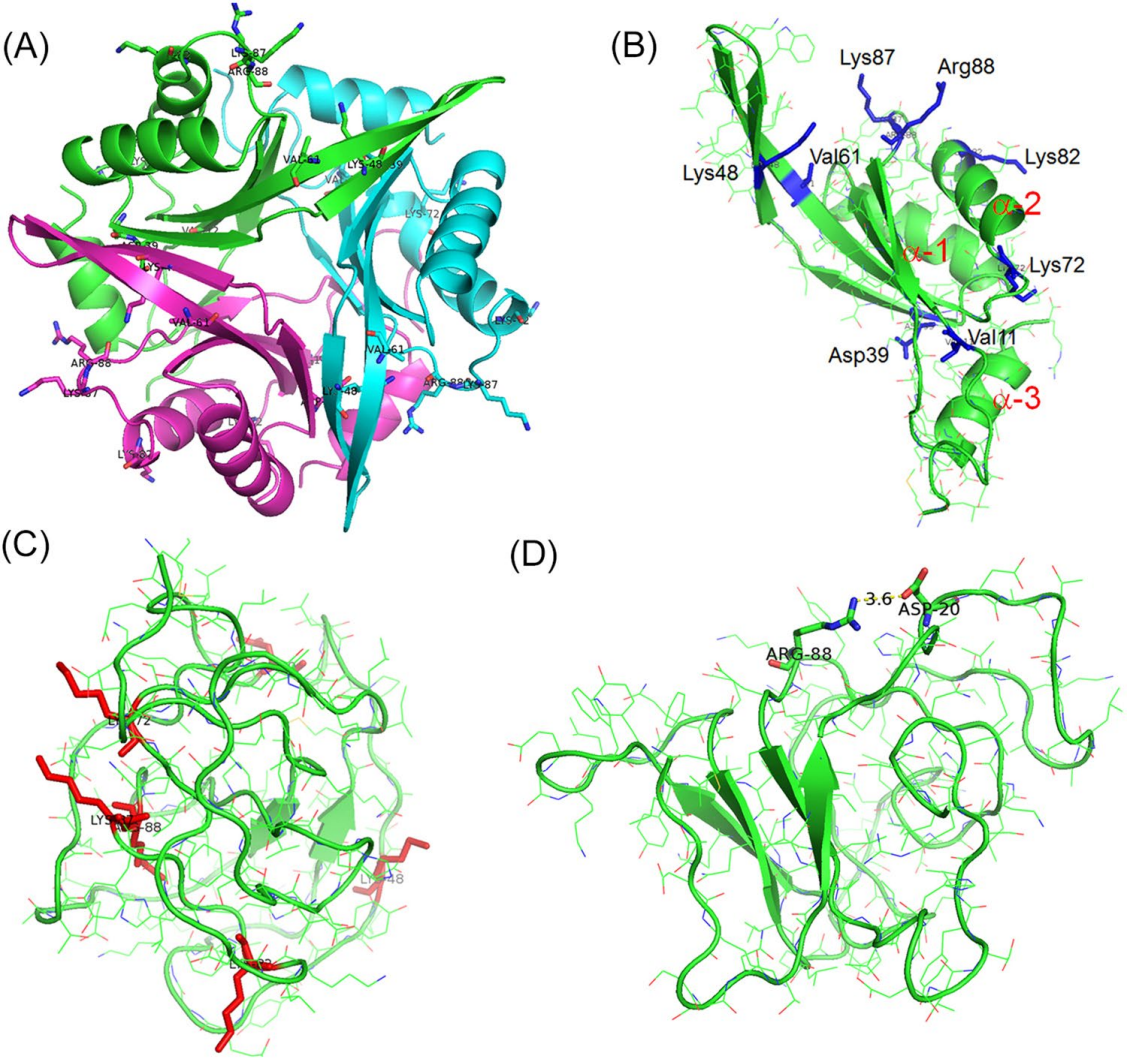
Tertiary structures of Ec0VV_6 during MD simulations. (**A**) Native trimer structure of Ec0VV_6 at 20 ns of 300 K MD simulation. Sticks represent targeted charged residues. (**B**) Monomer structure (A subunit) of Ec0VV_6 at 20 ns of 300 K MD simulation. Sticks represent targeted charged residues. Helices are numbered from the N-terminus (α-1, α-2, and α-3). (**C**) Monomer structure (C subunit) of Ec0VV_6 at 500 ns of 450 K MD simulation. Red sticks represent targeted charged residues. (**D**) Monomer structure (C subunit) of Ec0VV_6 at 1254.4 ns of 450 K MD simulation. The formation of an ion pair (3.6 Å) between Asp20 and Arg88 is shown.

The tertiary structure of EcCutA1 clearly resembles that of PhCutA1. The monomer structure consists of three α-helices and five β-strands, and three monomers are assembled into a trimer through interactions between the edges of three β-strands (Fig. 1A). We performed 1.4-μs MD simulations at 400 K and 450 K for three monomers (112 residues) of the described EcCutA1 mutants, and the structure ensembles in the heat-denatured state were evaluated by examining changes in features of the structure due to substitution of residues. The average occupancy of salt bridges by the six substituted residues of Ec0VV_6 was 89.5% at 450 K, indicating that about 90% of each charged residue forms salt bridges even in the heat-denatured ensemble. It appears that stabilization due to salt bridges in the native state is largely cancelled when salt bridges form in the denatured state. However, this interaction might suppress the flexibility of the protein in the denatured states, contributing to protein stability by decreasing entropy in the denatured state, similar to the mechanism responsible for stabilization of protein disulfide bonds^18^.

## Results

**Figure 2:**
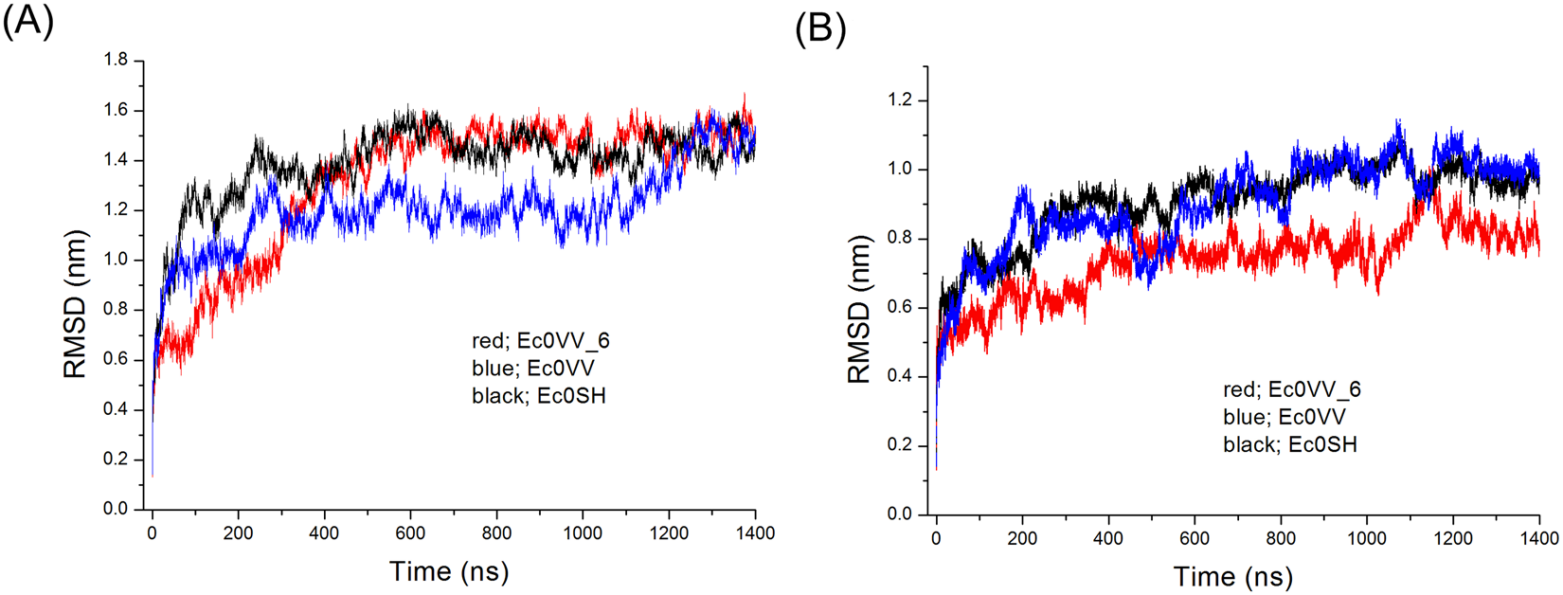
Trajectories of the average RMSD of Cα atoms of the three mutants over 1400 ns. (**A**) At 450 K. Red, blue, and black represent Ec0VV_6, Ec0VV, and Ec0SH, respectively. Each trajectory is the average of three subunits. (**B**) At 400 K. Red, blue, and black represent Ec0VV_6, Ec0VV, and Ec0SH, respectively. Each trajectory is the average of three subunits.

**Figure 3:**
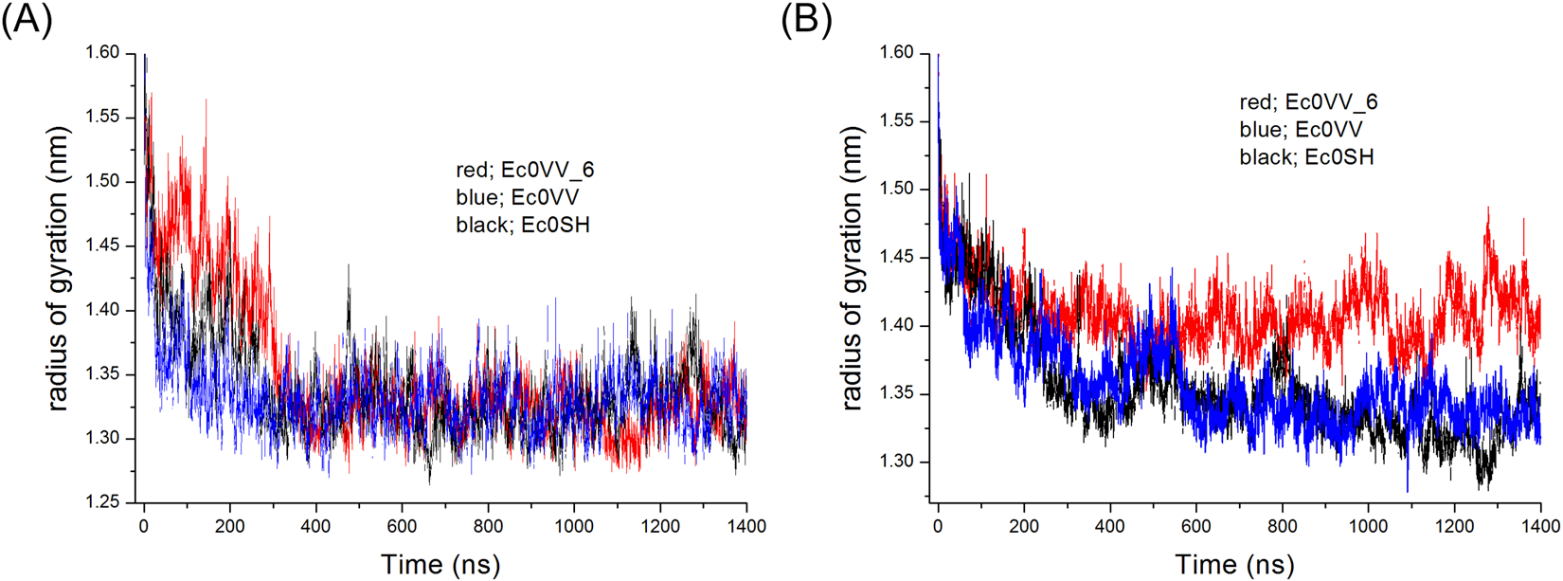
Trajectory of the average radius of gyration of the three mutants over 1400 ns. (**A**) At 450 K. Red, blue, and black represent Ec0VV_6, Ec0VV, and Ec0SH, respectively. Each trajectory is the average of three subunits. (**B**) At 400 K. Red, blue, and black represent Ec0VV_6, Ec0VV, and Ec0SH, respectively. Each trajectory is the average of three subunits.

We performed 1.4-μs MD simulations at 400 K and 450 K for each monomer (subunits A, B, and C) of three mutants (Ec0SH, Ec0VV, and Ec0VV_6). As a reference, 0.4-μs MD simulations at 300 K were also done for the native trimer structures of the three mutants. We found that all three mutant proteins are largely disordered and the average root mean square deviations (RMSDs) of all the Cα atoms reached a plateau after 200–400 ns simulations, at 400 K and 450 K (Fig. 2). The trajectories of the average radius of gyration for three mutant subunits are also shown in Fig. 3. The decrease in radius of gyration for all mutant monomers might be due to changes in structure, from ellipsoid in the native trimer (Fig. 1B) to globular in the disordered monomer (Fig. 1C). The average values of RMSD and radius of gyration are listed in Table S1. The average values of RMSD for each subunit at 450 K were greater than those at 400 K, suggesting that the structures at 450 K are more disordered.

**Figure 4:**
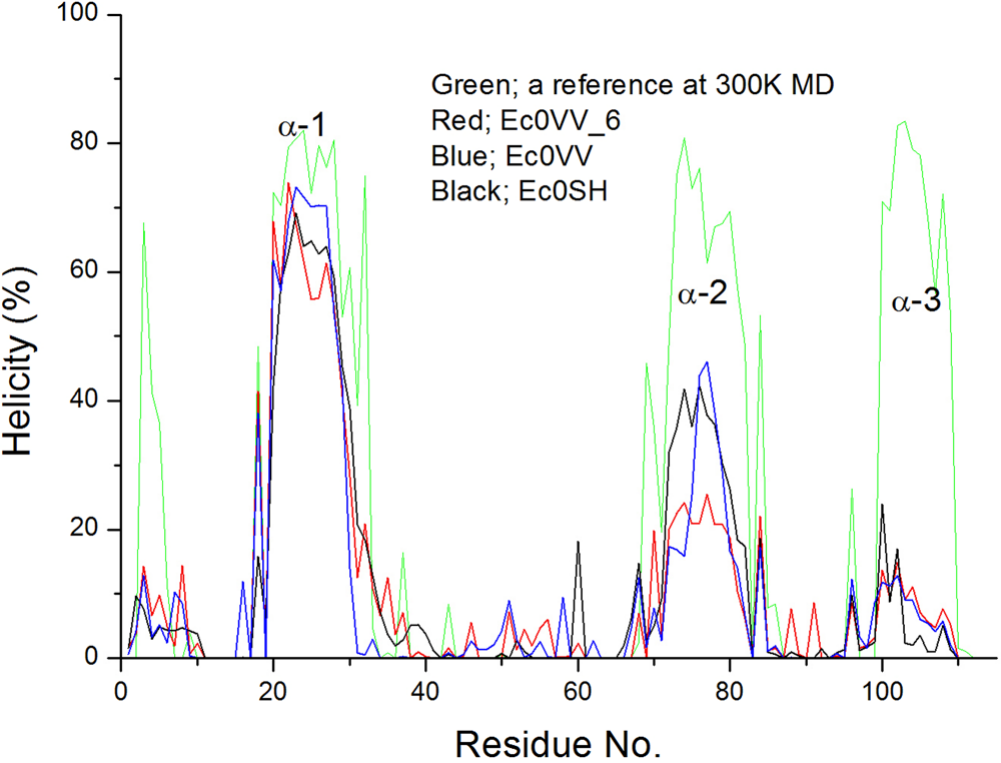
Average helicity of three mutants over 200 ns (1200–1400 ns) at 400 K MD. Red, blue, and black represent Ec0VV_6, Ec0VV, and Ec0SH, respectively. Green is a reference (Ec0VV_6) at 300 K.

At 450 K, the α-helical structures of all three mutants were nearly destroyed after 0.4 μs (Figure S1A, Table 1), but about half of the β-sheets were sustained during 1.4 μs (Table 1). By contrast, at 400 K, all three mutants retained approximately 40% of their α-helical structures during 1.4-μs simulation (Figure S1C, Table 1). Most of the α-helical residues sustained between 1.2–1.4 μs were seen in the α-1 helix in the trimer structure at 300 K (Fig. 4). Over the interval of 0.4–1.4 µs, average values of β-sheet for the three mutants were similar at 300 K and 400 K, but reduced by approximately half at 450 K (Table 1).

**Table 1:** Changes in the residue number of secondary structures in CutA1 mutants during 1.4-μs MD simulation.

Temperature	interval (ns)	Ec0VV_6			Ec0VV			Ec0SH		
		structure*	β-sheet	α-helix	structure*	β-sheet	α-helix	structure*	β-sheet	α-helix
300 K	50–200	85.5 ± 1.8	43.0 ± 1.6	35.4 ± 1.0	81.6 ± 1.9	40.0 ± 1.7	34.0 ± 0.9	82.3 ± 2.0	39.1 ± 2.0	34.6 ± 1.2
400 K	400–1400	66.6 ± 5.1	42.3 ± 3.6	15.6 ± 3.1	64.9 ± 4.2	37.8 ± 4.2	16.3 ± 3.3	65.2 ± 3.8	38.4 ± 3.3	16.2 ± 3.1
	1000–1400	63.6 ± 4.0	40.9 ± 3.3	13.5 ± 1.8	64.9 ± 4.5	39.9 ± 4.6	13.7 ± 1.7	63.7 ± 3.4	38.5 ± 3.2	14.4 ± 1.8
450 K	400–1400	39.8 ± 4.9	21.2 ± 4.5	1.2 ± 1.8	45.1 ± 4.8	24.0 ± 4.2	2.7 ± 1.7	41.5 ± 5.1	23.0 ± 5.1	1.0 ± 1.3
	1000–1400	38.7 ± 4.7	19.7 ± 4.4	0.7 ± 1.1	45.4 ± 4.6	22.6 ± 4.0	2.4 ± 1.8	43.5 ± 4.5	24.8 ± 4.7	1.1 ± 1.3

*Structure = β-sheet + α-helix + β-bridge + turn.

Values represent the average residue number of secondary structures.

The energy of hydrophobic interaction (Δ*G*_HP_) for stabilization of a protein can be calculated from differences in the accessible surface area (*ASA*) of hydrophobic and hydrophilic atoms between the native and the denatured states, using the following equation^32,33^:

1 $${\rm{\Delta }}{G}_{{\rm{HP}}}({\rm{kJ}}/{\rm{mol}})=15.4{\rm{\Delta }}AS{A}_{\mathrm{non} \mbox{-} \mathrm{polar}}-2.6{\rm{\Delta }}AS{A}_{{\rm{polar}}}$$

Here, Δ*ASA*_non-polar_ and Δ*ASA*_polar_ represent the differences in *ASA* of non-polar and polar atoms of all residues, respectively, between the native and the denatured states. Table 2 shows average *ASA* values of hydrophobic (C and S) and hydrophilic (N and O) atoms for the three CutA1 mutants during MD simulation at different temperatures. Assuming that the structures obtained in the high temperature simulations (400 K or 450 K, 0.4–1.4 μs) correspond to the denatured ensembles, which are in equilibrium with folded structures under physiological conditions, hydrophobic energies of the three mutants were calculated (Table 3). The structure obtained from MD simulations at 300 K was assumed to represent the native state. The differences in hydrophobic energies between Ec0VV and Ec0SH were estimated to be 20.8 kJ/mol and 18.2 kJ/mol at 400 K and 450 K, respectively. These results quantitatively indicate how the hydrophobic mutant, Ec0VV, is stabilized by hydrophobic interactions as compared with its template mutant, Ec0SH. On the other hand, the calculated hydrophobic energies of the ionic mutant substituted with six charged residues, Ec0VV_6, were approximately equal to those of its template mutant, Ec0VV (Table 3), indicating that the alkyl groups of charged residues do not contribute significantly to stabilization due to hydrophobic interaction.

**Table 2:** Average ASA values of hydrophobic and hydrophilic atoms for CutA1 mutants during MD simulations at different temperatures.

		400 K	450 K	300 K	Δ400	Δ450
Ec0VV_6	hydrophobic (C, S)	33.41 ± 8.22	34.42 ± 7.70	21.88 ± 4.17	11.53	12.54
	hydrophilic (N, O)	30.64 ± 5.09	30.21 ± 5.58	22.45 ± 3.40	8.18	7.76
Ec0VV	hydrophobic	32.06 ± 11.09	33.60 ± 7.26	20.69 ± 4.92	11.37	12.91
	hydrophilic	29.81 ± 7.17	30.19 ± 5.48	22.05 ± 4.18	7.76	8.15
Ec0SH	hydrophobic	31.49 ± 9.16	33.23 ± 7.98	21.54 ± 6.40	9.95	11.68
	hydrophilic	29.71 ± 7.07	30.28 ± 5.68	22.38 ± 5.00	7.33	7.90

All values are given in nm^2^.

ASA values at 400 K and 450 K are average values obtained between 0.4 μs and 1.4 μs. ASA values at 300 K are average values obtained between 0.05 μs and 0.4 μs. Δ400 or Δ450 is the difference between the ASA values at 300 K and 400 K or 450 K, respectively.

**Table 3:** Calculation of hydrophobic energy assuming that the ASA values at 400 K or 450 K represent those of the denatured states.

	400 K	450 K	Δ400_1_	Δ400_2_	Δ450_1_	Δ450_2_
Ec0VV_6	156.3	172.9	22.2	1.4	13.6	−4.7
Ec0VV	155.0	177.6	20.8		18.2	
Ec0SH	134.1	159.4				

Calculations were performed using the ΔASA values listed in Table 2 using equation(1). All values are given in kJ/mol.

Subscript 1 indicates the calculated difference between the listed mutant and Ec0SH; subscript 2 indicates the difference between the listed mutant and Ec0VV.

**Figure 5:**
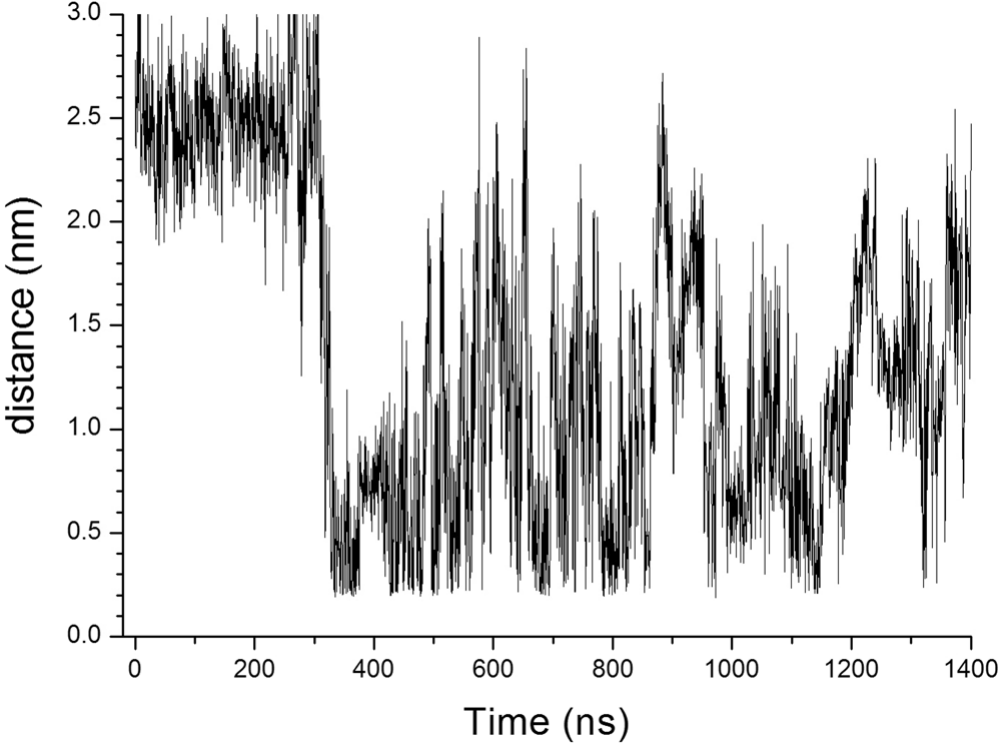
Typical trajectory of changes in the distance between charged residues during MD simulation The figure shows changes in distance between the Cγ atom of Asp39 and the Cε atom of Lys87 in the Ec0VV_6 B subunit over 1400 ns at 450 K. Other examples are shown in Figure S2 and Figure S3.

**Figure 6:**
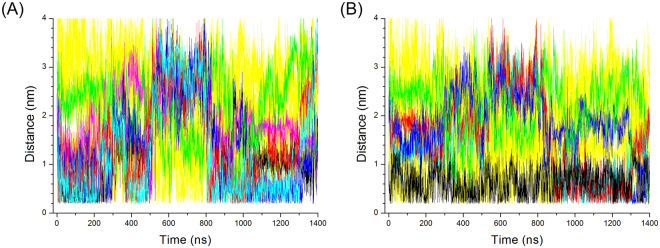
Trajectories of changes in the distance of salt bridges of Lys87 and Arg88 in Ec0VV_6A during 1400 ns at 450 K. (**A**) Salt bridges of Lys87: Lys87–Asp20 (black), Lys87–Asp26 (magenta), Lys87–Asp39 (green), Lys87–Glu21 (blue), Lys87–Glu57 (red), Lys87–Glu59 (cyan), and Lys87–others (yellow). (**B**) Salt bridges of Arg88: Arg88–Asp20 (cyan), Arg88–Glu21 (red), Arg88–Asp26 (blue), Arg88–Asp39 (green), Arg88–Glu90 (black), and Arg88–others (yellow).

**Figure 7:**
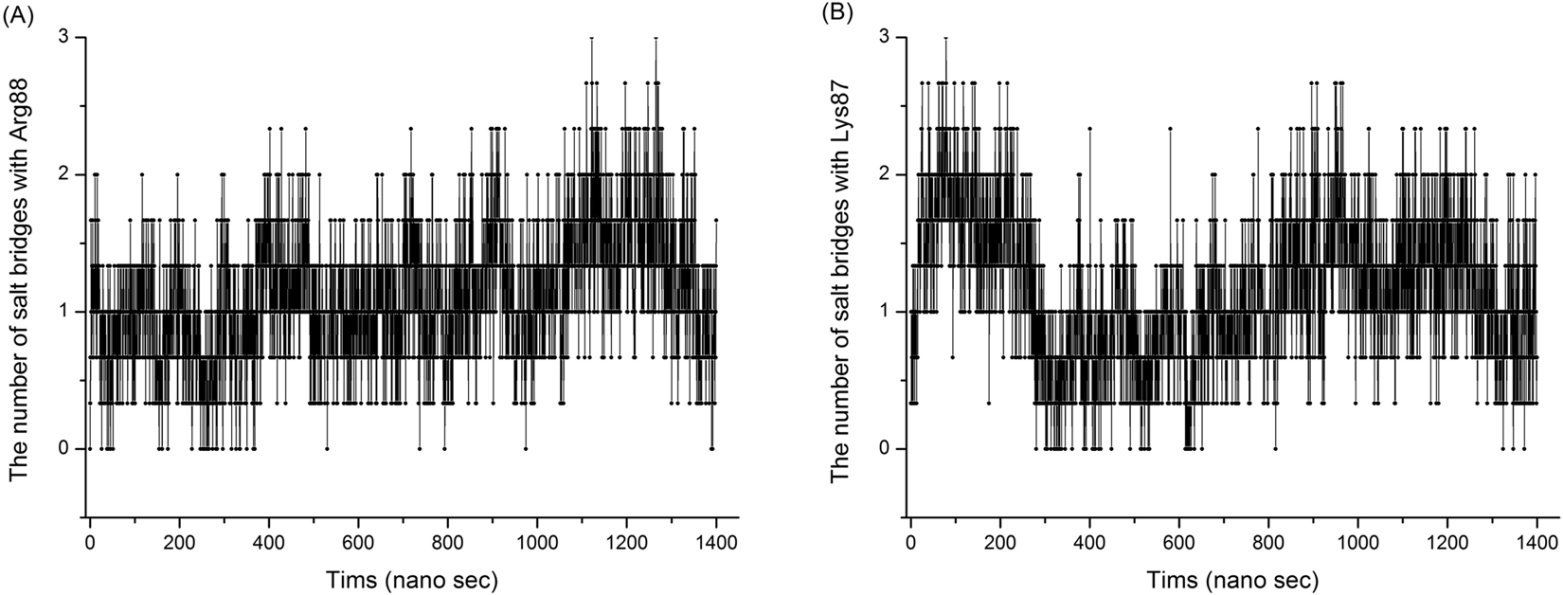
Trajectory of the number of salt bridges with Arg88 and Lys87 in Ec0VV_6 over 1400 ns at 450 K. (**A**) and (**B**) represent Arg88 and Lys87, respectively. The data are averages for three subunits of Ec0VV_6A, B, and C, calculated every 0.4 ns. A line and symbol were drawn every 0.4 ns. A salt bridge was counted when the distance of a favorable ion–ion interaction was less than 0.6 nm. In a 1.4-μs MD simulation performed at 450 K, the average number of salt bridges with Arg88 and Lys 87 was 1.12 and 1.15, respectively.

Two charged residues may form a salt bridge when the distance between Cε or Cδ of a positively charged residue (Lys or Arg) and Cδ or Cγ of a negatively charged residue (Glu or Asp) is less than 0.6 nm^27^. Figure 5 shows the typical trajectory of changes in distance between Cε of Lys87 and Cγ of Asp39 in Ec0VV_6B during 1.4-μs simulations at 450 K. Lys87 can probably make a salt bridge with Asp39 during this simulation, although the distance between the two residues is greater than 2.5 nm in the native structure. The interaction frequency of Lys87 and Arg88 with other favorable negatively charged residues is shown in Figs 6A and B, respectively. These individual interactions with Lys87 and Arg88 are also shown separately in Figures S2 and S3. As the figures show, each favorable salt bridge fluctuated drastically during simulations, but distance is less than 0.6 nm (suitable for formation of salt bridges) for a considerable portion of the time in both plots. As shown in Fig. 7, approximately one salt bridge with Arg88 (or Lys87) was preserved during MD simulations even at 450 K, even though favorable pair residues with Arg88 (or Lys87) frequently replace during simulations. The average number of salt bridges with Arg88 and Lys 87 was 1.12 ± 0.45 and 1.15 ± 0.53, respectively, during 1.4-μs MD simulations at 450 K, respectively. Furthermore, the occupancy of salt bridges by targeted charged residues during MD simulations could be calculated from the trajectory of the changes in distance between atoms of favorable ion pairs (Fig. 5). Table 4 shows the occupancies of salt bridges by the six substituted residues of Ec0VV_6 during MD simulation at 450 K, 400 K, and 300 K. An occupancy value of more than 100% indicates that a charged residue forms more than one salt bridge. The occupancies of salt bridges by the six substituted residues of Ec0VV_6 were 72.9–120.9% and 23.2–130.3% at 450 K and 400 K, respectively, compared to 61.7–285.4% at 300 K. The average percent occupancies were 89.5%, 84.3%, and 140.1% at 450 K, 400 K, and 300 K, respectively; thus, the average occupancy of salt bridges at 450 K was approximately 60% of the occupancy at 300 K. The data in Table 4 also shows how the pairs of native-state salt bridges at 300 K were replaced at 400 K and 450 K during simulations. These results suggest that many charged residues in proteins from hyperthermophiles also form a high percentage of salt bridges in the denatured state.

**Table 4:** Percent occupancy of ion pairs (less than 0.6 nm) for substituted residues of Ec0VV_6 at 450 K, 400 K, and 300 K during MD simulations.

	substituted residue	pair residue	450 K	400 K	300 K		substituted residue	pair residue	450 K	400 K	300 K		substituted residue	pair residue	450 K	400 K	300 K
(A)	Arg88	Asp3	3.0	0.6		(C)	Lys48	Asp3	1.6			(E)	Lys82	Asp3	4.0	0.0	
		Glu4	2.5	0.4				Glu4	1.9					Glu4	3.2	0.9	
		Asp20	20.5					Asp20	2.1					Asp20	3.0	2.4	
		Glu21	19.1					Glu21	4.5					Glu21	3.4		
		Asp26	5.1					Asp26	4.7					Asp26	5.9	10.6	
		Glu34	3.3					Glu34	5.3					Glu34	1.0	7.4	8.8
		Asp39	6.8		4.9			Asp39	6.4		97.4			Asp39	4.2		
		Glu53	2.5	48.5				Glu53	4.3					Glu53	11.1	3.6	
		Glu57	1.4	3.0				Glu57	11.7		2.3			Glu57	4.8		
		Glu59	3.2	0.3				Glu59	10.2	0.2	85.8			Glu59	3.9	0.1	
		Glu78	1.6	3.1	0.4			Glu78	1.4					Glu78	13.0	49.5	81.8
		Glu90	49.5	46.2	7.7			Glu90	6.4	17.4	100.0			Glu90	3.6	1.6	
		Asp100	0.1		23.4			Asp100	8.0	2.9				Asp100	6.5		
		Asp102	0.6		25.3			Asp102	6.3	2.3				Asp102	8.7		
		C-terminal	1.9	1.1				C-terminal	2.5	0.5				C-terminal	4.3	4.3	
	sum		120.9	103.2	61.7		sum		77.4	23.2	285.4		sum		80.7	80.4	90.7
	**substituted residue**	**pair residue**	**450 K**	**400 K**	**300 K**		**substituted residue**	**pair residue**	**450 K**	**400 K**	**300 K**		**substituted residue**	**pair residue**	**450 K**	**400 K**	**300 K**
**(B)**	Asp39	N-terminal	5.1	1.1		**(D)**	Lys72	Asp3	5.6	18.7		**(F)**	Lys87	Asp3	4.3	0.2	
		Lys5	1.0	1.4				Glu4	11.9	21.1	0.7			Glu4	4.4	0.3	
		Lys30	7.2					ASp20	3.2					Asp20	13.6	34.6	15.8
		Lys35	8.1	2.7				Glu21	3.2					Glu21	20.6	0.8	
		Lys48	6.4		97.4			Asp26	1.9					Asp26	6.9	0.8	
		Lys55	1.9					Glu34	9.5	20.4				Glu34	4.4		
		Lys67	16.0	87.2	100.0			Asp39	5.2	0.1				Asp39	8.5		
		Lys72	5.2	0.1				Glu53	2.8					Glu53	3.9	20.9	
		Lys81	1.4					Glu57	9.0					Glu57	10.8	23.7	30.8
		Lys82	4.2					Glu59	8.8					Glu59	19.7	48.6	41.3
		Lys87	8.5					Glu78	3.2	3.4				Glu78	5.6	0.1	
		Arg88	6.8		4.9			Glu90	0.8	0.0				Glu90	3.9	0.3	15.3
		Arg112	1.1	4.3				Asp100	3.4	2.3				Asp100	0.7		
								Asp102	1.8	4.2				Asp102	0.4		0.9
								C-terminal	4.4	2.0	95.6			C-terminal	3.1		
	sum		72.9	96.8	202.3		sum		74.5	72.2	96.3		sum		110.8	130.3	104.1

The values at 450 K and 400 K were taken during 1000 ns of MD between 0.4 µs and 1.4 µs, and represent an average of three subunits. Values at 300 K were obtained between 0.05 µs and 0.4 µs.

## Discussion

There are several reports^4–6,34,35^ focusing on the denatured state ensembles that are in equilibrium with the native state under physiological conditions. Denatured state ensembles may be considerably compact, similar to the molten globule state, containing secondary structures^36^ that are not suited to the random-coil model^37^. Denatured structures of pyrrolidone carboxyl peptidase under physiological conditions have been reported to form non-native hydrophobic clusters and native-like helices^5,6^. In the denatured state, charged residues are also involved in strong electrostatic interactions^3,34,38^.

In the current study, we performed 1.4-μs MD simulations of EcCutA1 stability-enhanced mutants at 400 K and 450 K to elucidate characteristics of denatured state ensembles. For the Ec0VV_6 mutant, at 450 K nearly all α-helices and β-sheets disappeared transiently at 500 ns (Fig. 1C), but on average 20% of the total residues maintained β-sheets during the period of 0.4–1.4 μs (Table 1), although the RMSD suggests that the conformation of Ec0VV_6 is drastically disordered (Table S1). At 400 K, one third of the α-helical content of Ec0VV_6 was maintained even after 1.0–1.4 μs simulation (Table 1).

Proteins from hyperthermophiles contain considerably more charged amino acid residues than those of mesophiles^8^, and ion–ion interactions are believed to stabilize the proteins to permit growth at high temperatures^39^. The CutA1 protein from *Pyrococcus horikoshii* (PhCutA1) has many charged residues and is very stable, with a denaturation temperature of nearly 150 °C^20^. Although the electrostatic energy due to charged residues seems to stabilize PhCutA1 in the native state^12^, it has not been shown how these charged residues contribute to stabilization of the protein in the denatured state. Our salt bridge occupancy results at high temperatures suggest that charged residues form considerable numbers of favorable ion–ion interactions in the denatured state; salt bridge occupancies were 89.5% and 84.3% at 450 K and 400 K, respectively. These values were approximately 60% of the salt bridge occupancy value in the native state (300 K). It is difficult to determine whether the simulated ensembles at 400 K or 450 K are more representative of the denatured state ensembles under physiological conditions. However, the occupancies of ion–ion interactions were similar at 400 K and 450 K, although the secondary structure content was different. These results suggest that when sufficient charged residues are present, a considerable number of charged residues can make salt bridges in the denatured state ensemble. Favorable ion–ion interactions in the denatured state would stabilize the denatured conformation^40^, resulting in destabilization of the native protein. On the other hand, favorable ion–ion interactions in the denatured state ensemble could also decrease the entropy of the conformation in the denatured state, resulting in stabilization of the native protein. When one or two disulfide bonds are broken, a protein is substantially destabilized due to an increase in entropy in the denatured state^18^. In the current study, approximately 60% of the ion–ion interactions present at 300 K were maintained even at 450 K, suggesting that proteins from hyperthermophiles with a high ratio of charged residues are stabilized by decrease in conformational entropy due to ion–ion interactions in the denatured state.

Hyperthermophilic proteins differ greatly from mesophilic proteins in their change in heat capacity upon denaturation due to the presence of residual structure in the denatured state ensemble^16,41,42^. This feature results in increased protein stability at higher temperatures due to the broadening of temperature function curves for the Gibbs energy of unfolding. These results suggest that the denatured state conformation of hyperthermophilic proteins is more compact than that of mesophilic ones, indicating the role of entropy in determining stability. By analyzing thermodynamic data for 116 proteins, Sawle and Ghosh^43^ have reported that entropic stabilization is responsible for the high melting temperature of proteins observed in hyperthermophiles because the gain in enthalpy upon folding is smaller in hyperthermophiles than in mesophiles, whereas the loss of entropy upon folding is greater in mesophiles than in hyperthermophiles.

To our knowledge, it has not been reported that ion–ion interactions in the denatured state ensemble contribute to protein stability by decreasing conformational entropy, similar to the mechanism of stabilization by disulfide bonds. Introduction of charged residues for the formation of the salt bridges in the denatured state may be a useful tool for engineering proteins with improved conformational stability.

## Methods

MD simulations were performed using each subunit of three EcCutA1 mutants: an SH-free mutant with *T*_d_ = 85.6 °C (Ec0SH = EcCutA1_C16A/C39A/C79A), a hydrophobic mutant also lacking SH groups with *T*_d_ = 113.3 °C (Ec0VV = Ec0SH_S11V/E61V), and an ionic mutant with *T*_d_ = 136.8 °C (Ec0VV_6 = Ec0VV_A39D/S48K/H72K/S82K/Q87K/T88R)^23^.

MD simulations were performed using GROMACS software (ver. 4.5.5)^44,45^. The missing atoms in the coordinate file of Ec0SH (PDB ID, 4Y65), which are three N-terminal residues of the B subunit and eight N-terminal residues of the C subunit, were modeled in QUANTA2000 (Accelrys) using the coordinates of N-terminal residues of the A subunit as a reference. The structures of Ec0VV and Ec0VV_6 were modeled using FoldX, based on the structure of Ec0SH. Hydrogen atoms were added to each protein. The models were solvated in water boxes with a minimum distance of 1.2 nm between the protein and the box. Counter-ions were added to the model to neutralize any net charge. The periodic boundary condition was adopted and the long-range electrostatic interactions were computed using the Particle-Mesh-Eward (PME) method^46^. The GROMOS 43A1 force field and SPC/E water model^47^ were employed. The system was weakly coupled to a heat bath by velocity rescaling^48^ with a relaxation time of 0.1 ps. A Parrinello–Rahman barostat^49^ was used to maintain a pressure constant at 1.0 bar for 300 K and 6.0 bar for 400 K or 450 K with a relaxation time of 0.5 ps. Hydrogen atoms were constrained using LINCS^50^, and MD simulations at 300 K, 400 K, and 450 K were conducted with an integration time step of 1 femtosecond (fs). Energy minimizations were done to remove bad van der Waals contacts. Next, the temperature was raised from 50 K to 300 K in increments of 50 K, with 10,000 integration steps at each temperature and a harmonic constraint of C-alpha atoms. Thereafter, the ensemble was equilibrated through four 100-picosecond (ps) cycles with gradually released harmonic constraints: 1000, 100, 10, and 1 kJ mol^−1^ nm^−2^. The subsequent MD stages for the EcCutA1 mutants were carried out without any restraint at 300 K. When the system temperature was increased to 400 K or 450 K from 300 K, pressure coupling was not set during 1000 ps at 400 K or 450 K. The obtained MD trajectories were analyzed using GROMACS software. The calculations for RMSD of Cα atoms and the radius of gyration were performed using the commands ‘gmx rms’ and ‘gmx gyrate,’ respectively. For the salt bridges, ‘gmx saltbr’ was used with the option t = 0.4 nm, which means that groups that were never closer than this distance were not plotted. For the calculation of ASA values of each atom, the results of ‘gmx sasa’ (atomarea.xvg: average area per atom) were used. For the trajectory of secondary structures, the command ‘do_dssp’ was used. For the average α-helix at each residue, the command ‘g_helix’ was used.

## Electronic supplementary material

Supplementary Information
