# Supplementary material for: Ion–ion interactions in the denatured state contribute to the stabilization of CutA1 proteins
Source: Sci Rep. 2018 May 16;8:7613. doi: 10.1038/s41598-018-25825-7 (PMC5955972; doi:10.1038/s41598-018-25825-7)

**Supplemental Information**

Ion–ion interactions in the denatured state contribute to the stabilization of CutA1 proteins

Katsuhide Yutani^1,*^, Yoshinori Matsuura^1^, Hisashi Naitow^1^, and Yasumasa Joti^2^,

^1^RIKEN SPring-8 Center, 1-1-1 Kouto, Sayo, Hyogo 679-5148, Japan

^2^Japan Synchrotron Radiation Research Institute, 1-1-1, Kouto, Sayo, Hyogo 679-5198 Japan


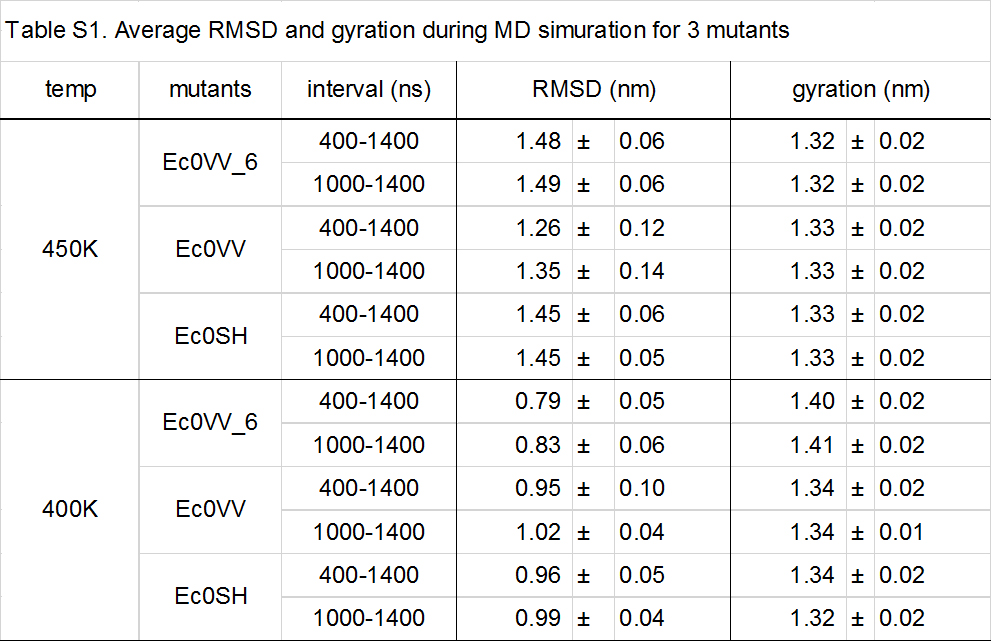


Supplementary figure legends

Figure S1. Trajectories of changes in secondary structures of three subunits during MD simulations

1. α-helical residues for three mutants at 450 K MD. Red, blue, and black represent Ec0VV_6, Ec0VV, and Ec0SH, respectively. The values are an average of all three subunits.
2. Structural residues (= β-sheet + α-helix + β-bridge + turn) for three mutants at 450 K MD. Red, blue, and black represent Ec0VV_6, Ec0VV, and Ec0SH, respectively. The values are an average of all three subunits.
3. α-helical residues for 3 mutants at 400 K MD. Red, blue, and black represent Ec0VV_6, Ec0VV, and Ec0SH, respectively. The values are an average of all three subunits.
4. Structural residues (= β-sheet + α-helix + β-bridge + turn) for three mutants at 400 K MD. Red, blue, and black represent Ec0VV_6, Ec0VV, and Ec0SH, respectively. The values are an average of all three subunits.

Figure S2. Trajectories of changes in the distance of major salt bridges of Lys87 in Ec0VV_6A during 1400 ns at 450 K

1. Lys87–Asp20 (black)
2. Lys87–Asp26 (magenta)
3. Lys87–Asp39 (green)
4. Lys87–Glu21 (blue)
5. Lys87–Glu57 (red)
6. Lys87–Glu59 (cyan)

Figure S3. Trajectories of changes in the distance of major salt bridges of Arg88 in Ec0VV_6A during 1400 ns at 450 K

1. Arg88–Asp20 (cyan)
2. Arg88–Glu21 (red)
3. Arg88–Asp26 (blue)
4. Arg88–Asp39 (green)
5. Arg88–Glu90 (black)


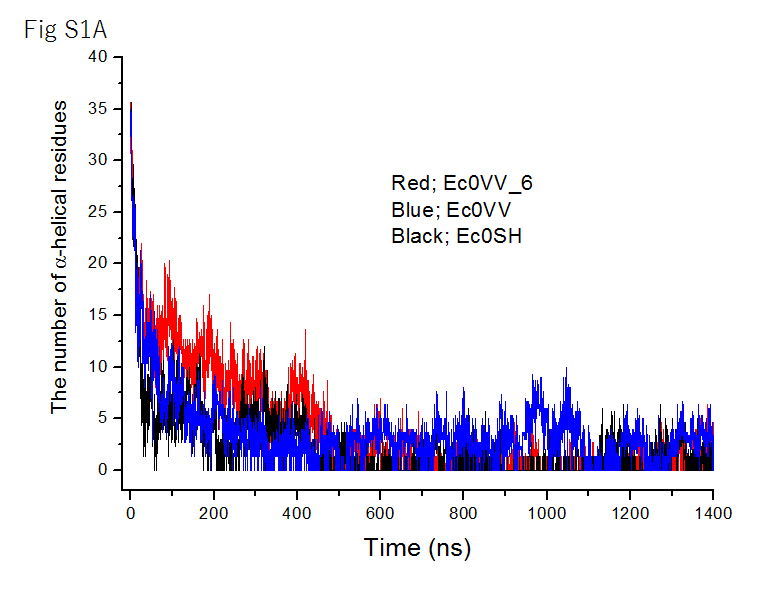


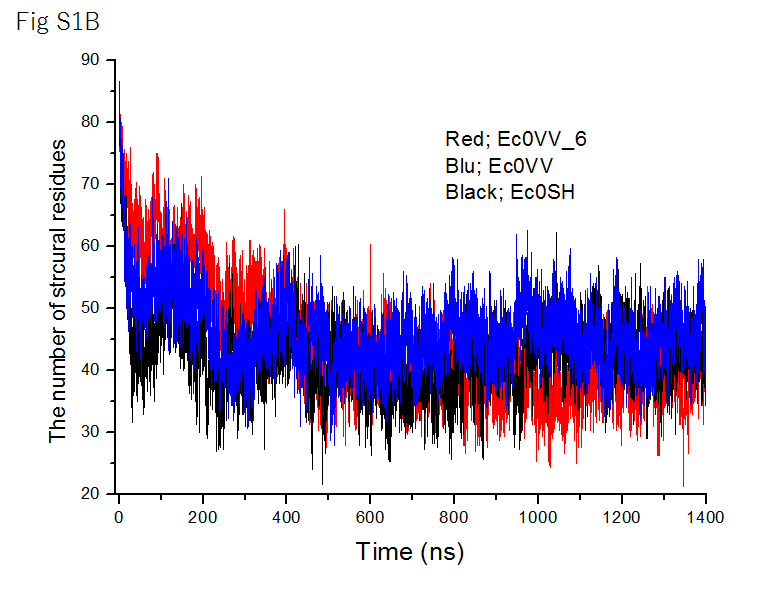


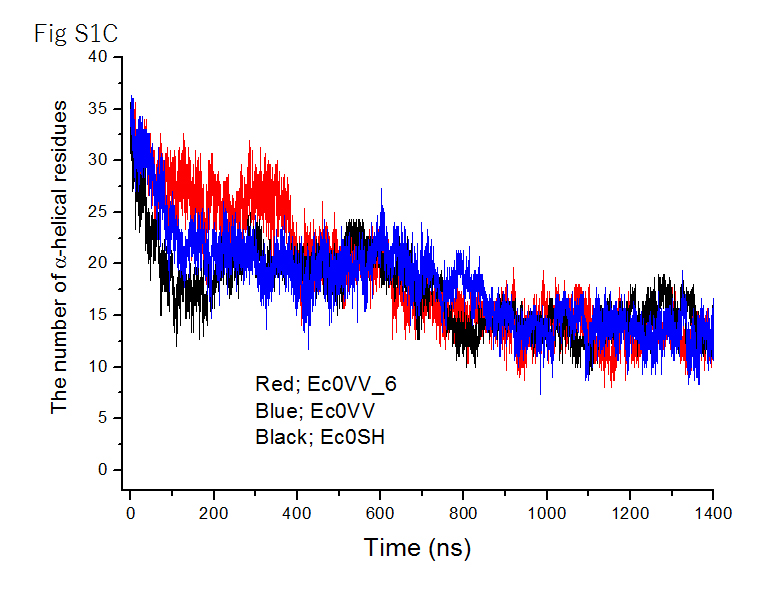


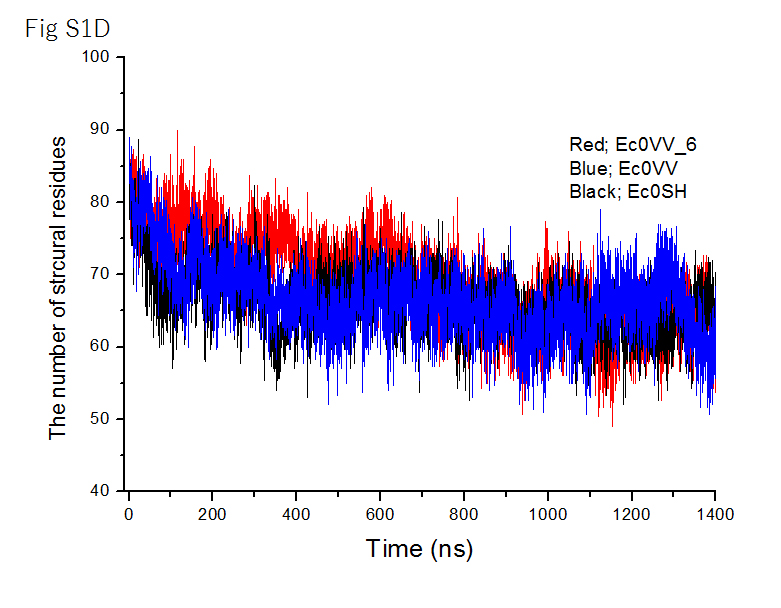


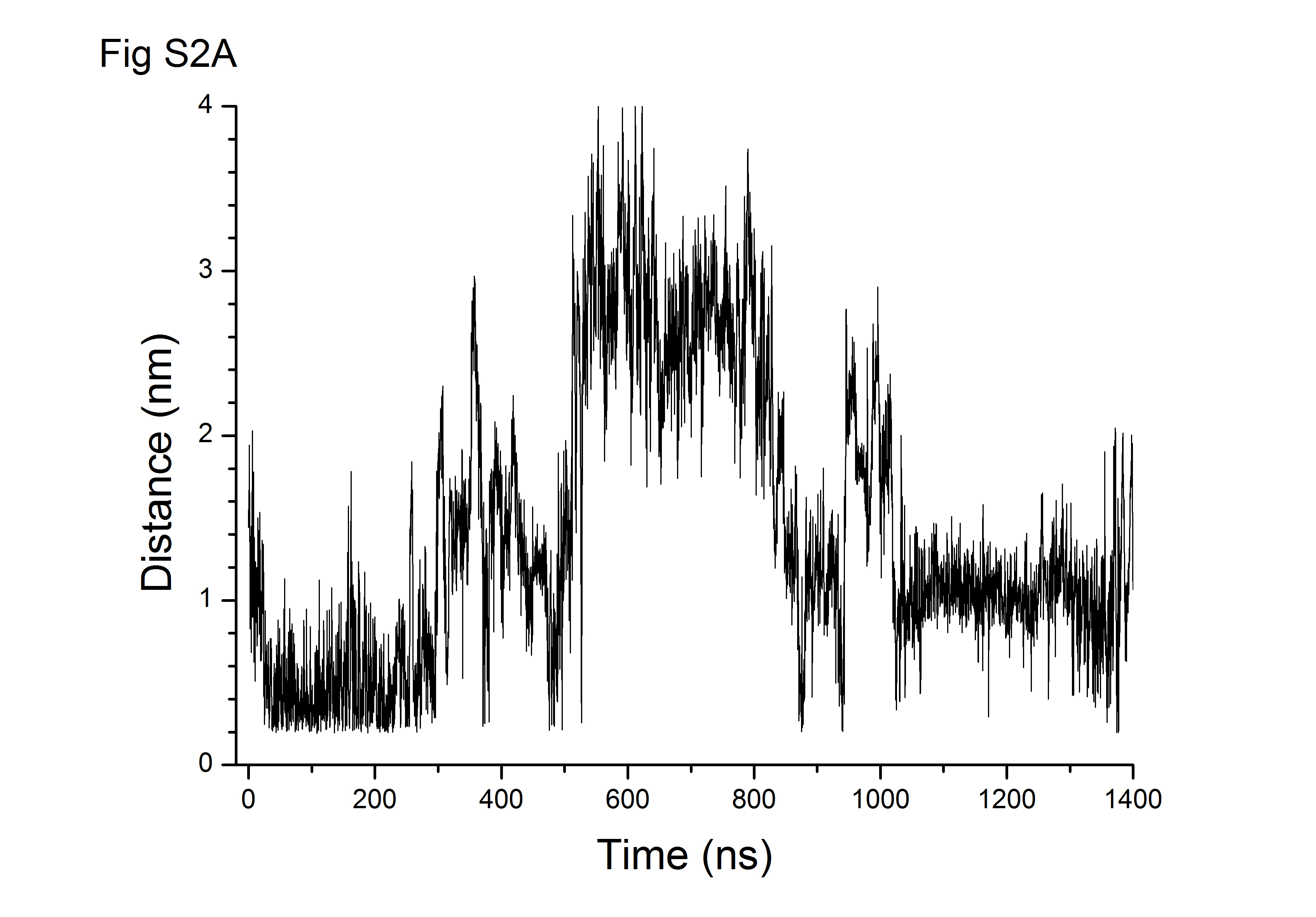


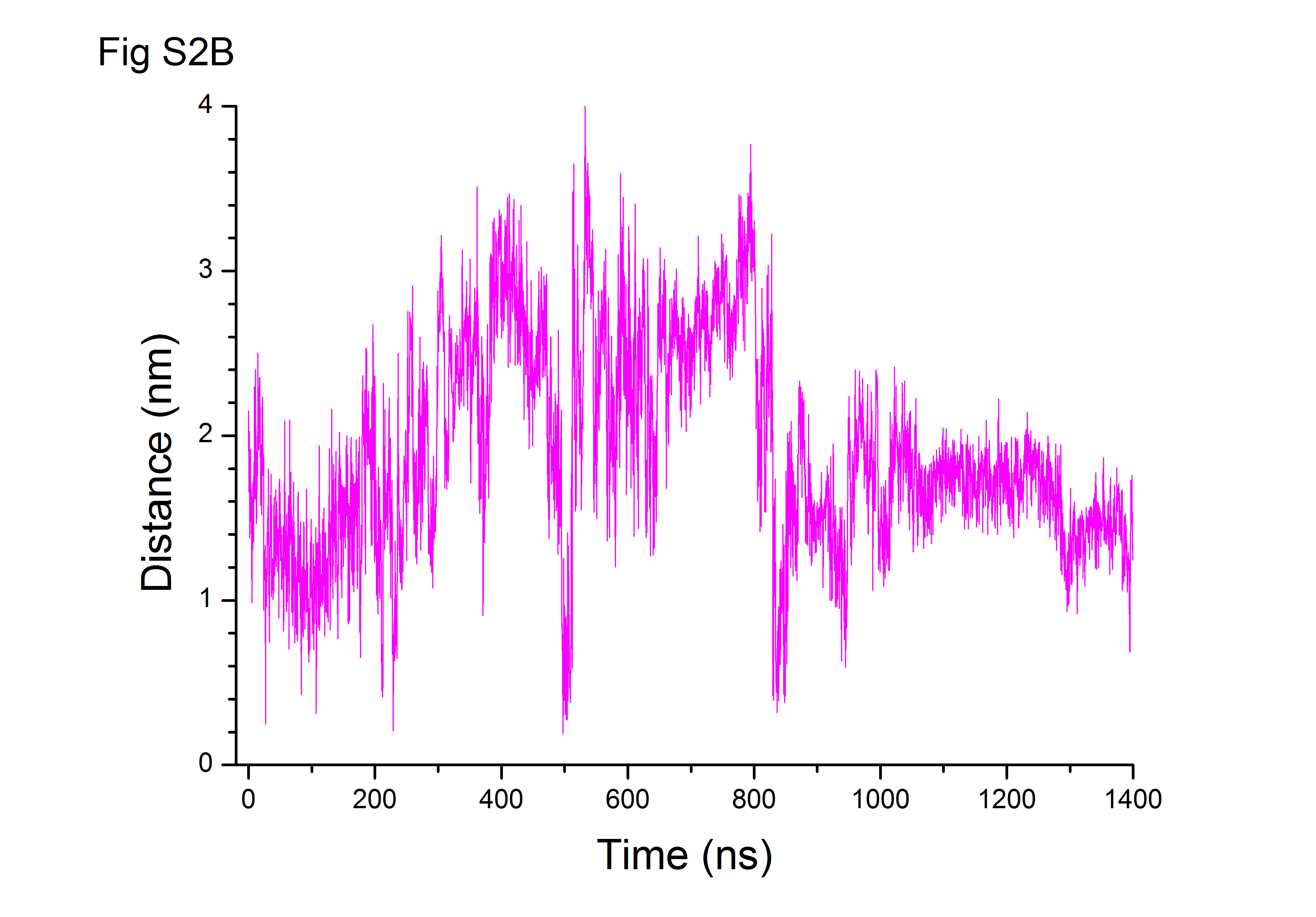


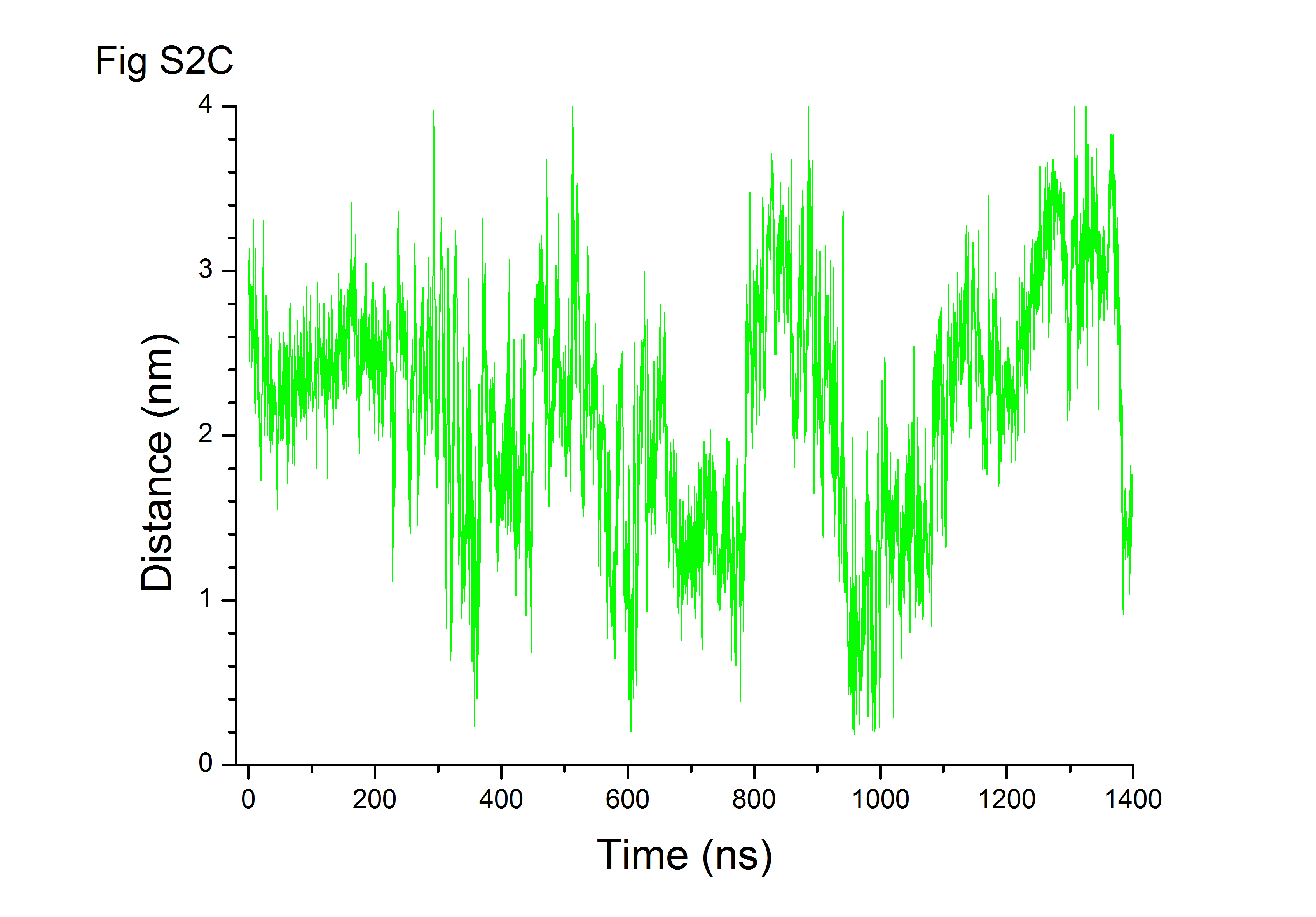


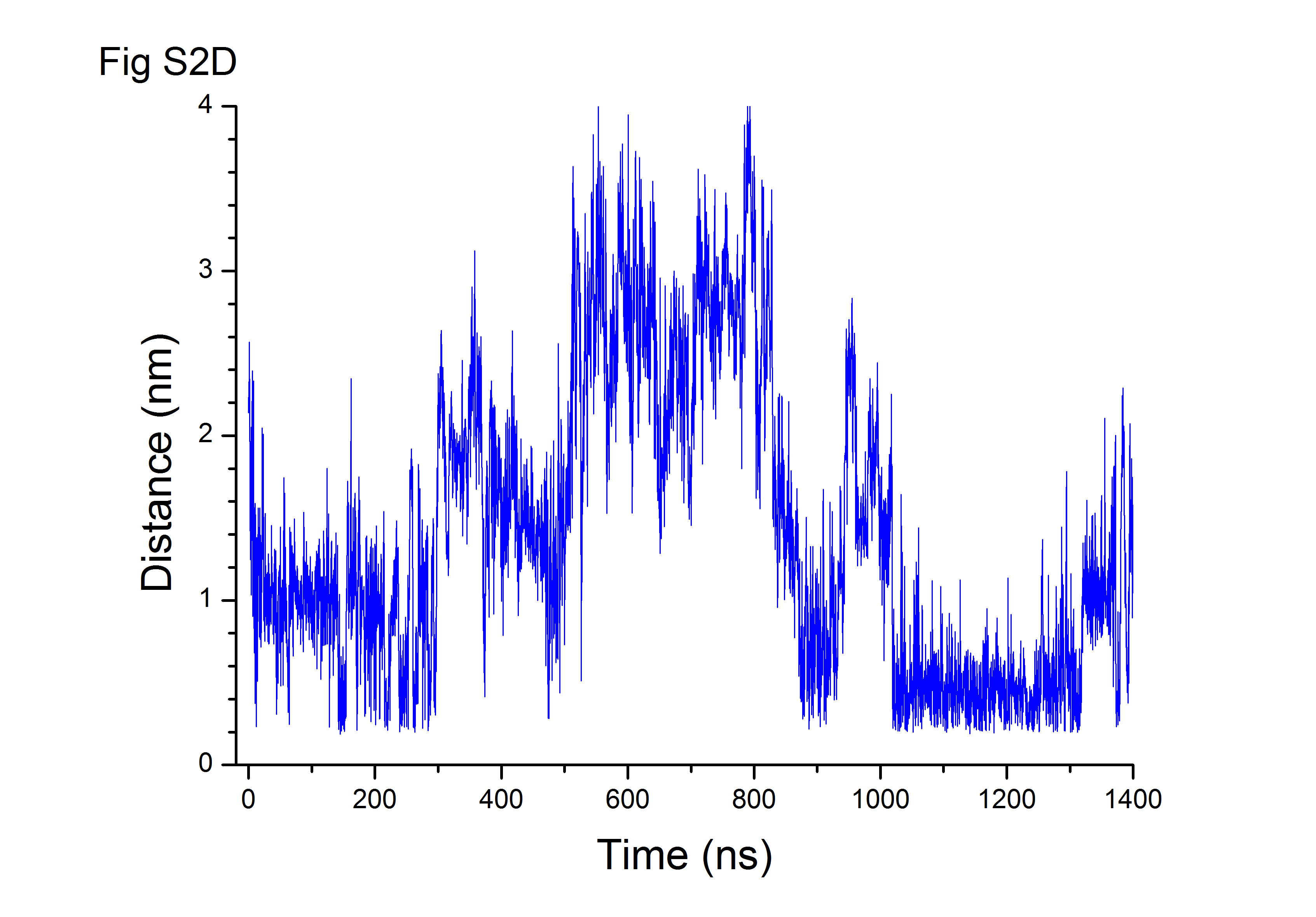


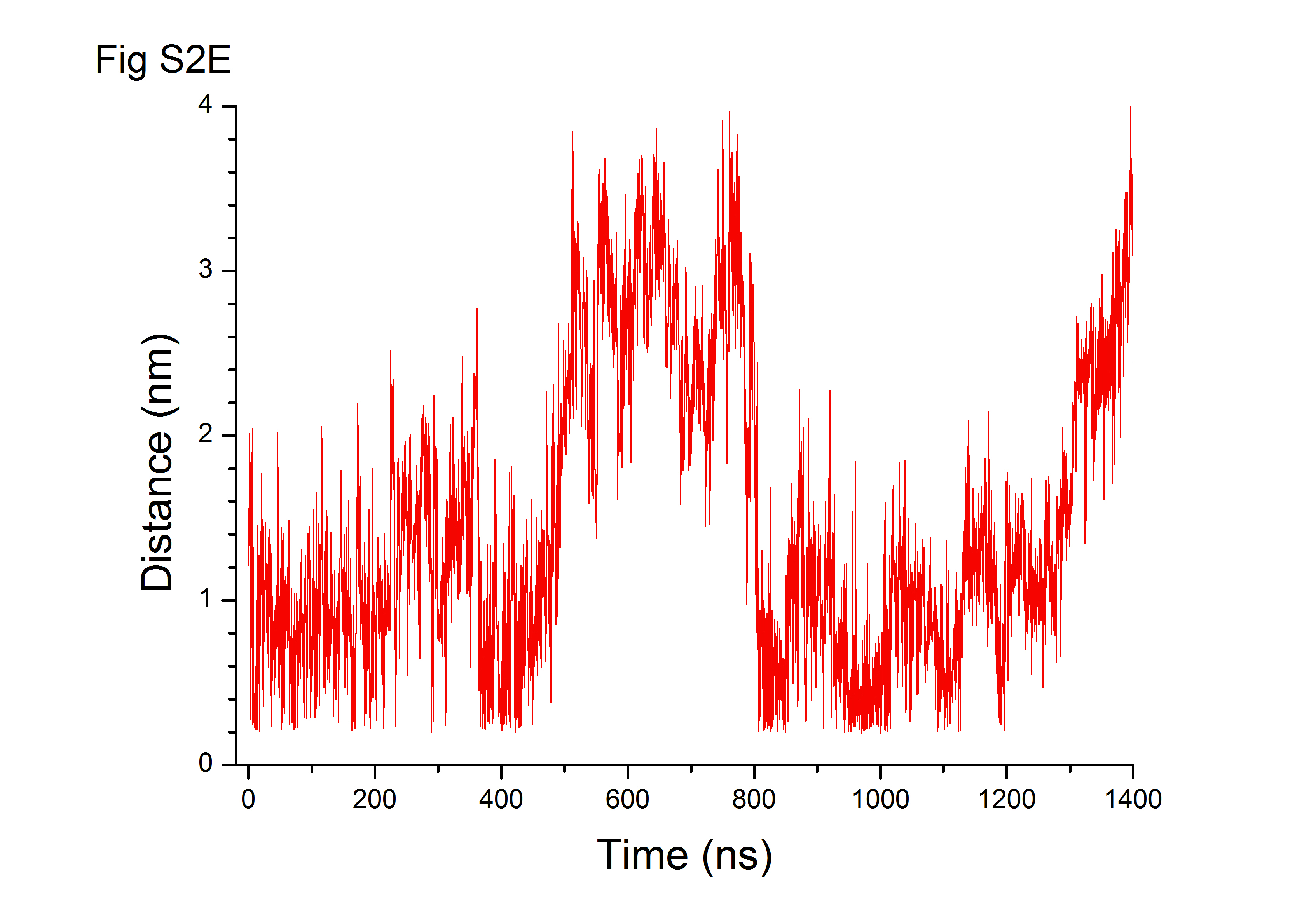


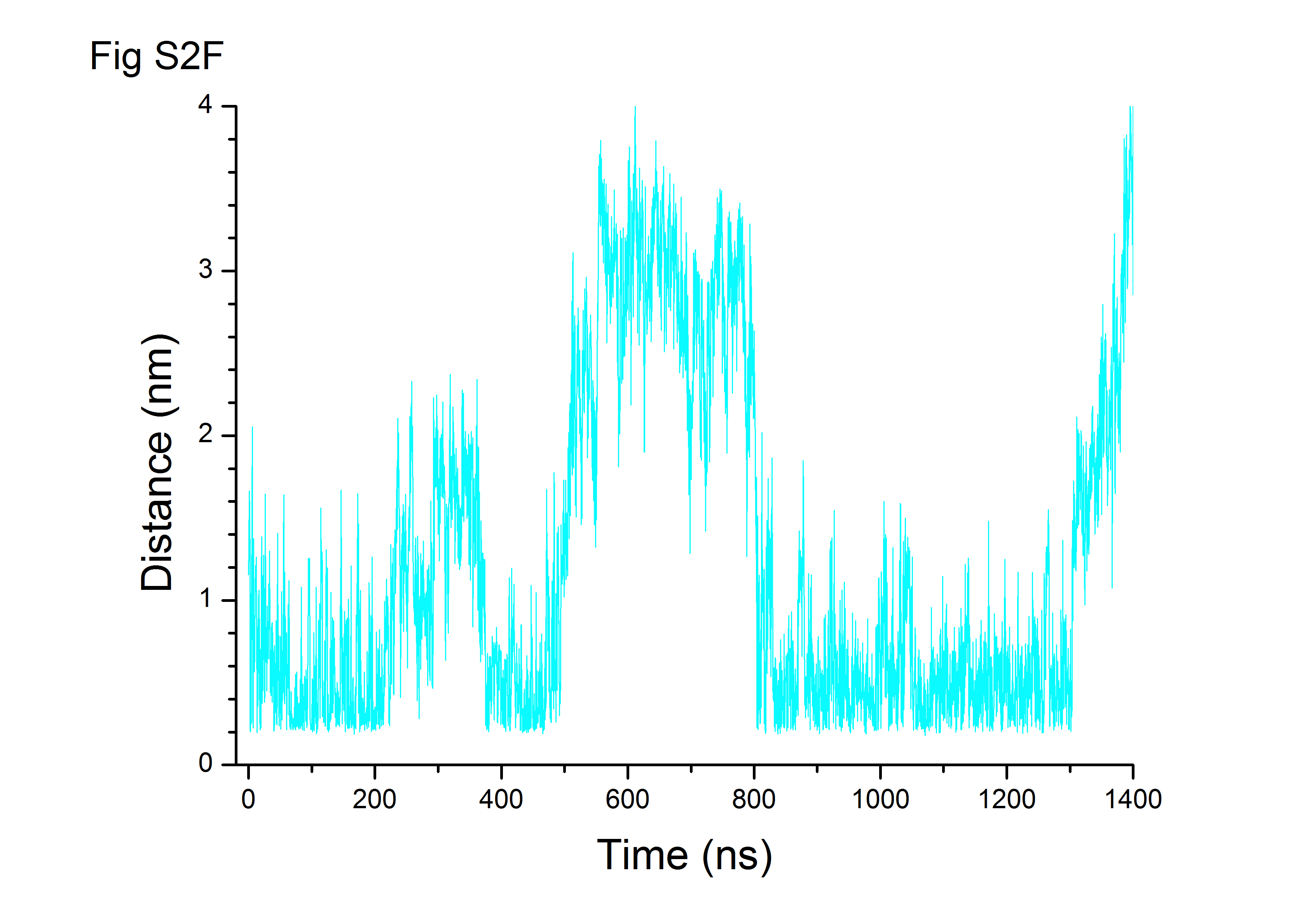


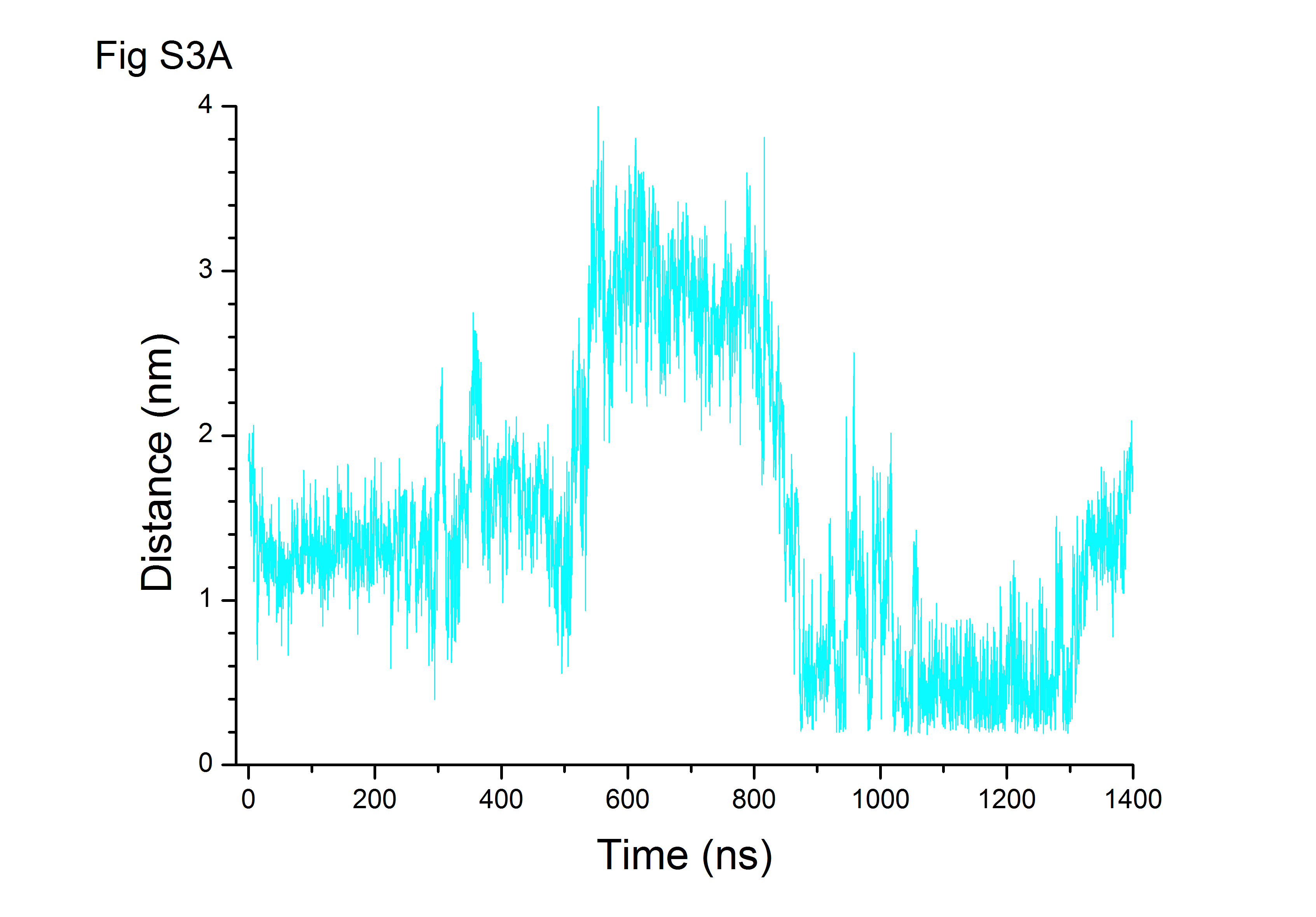


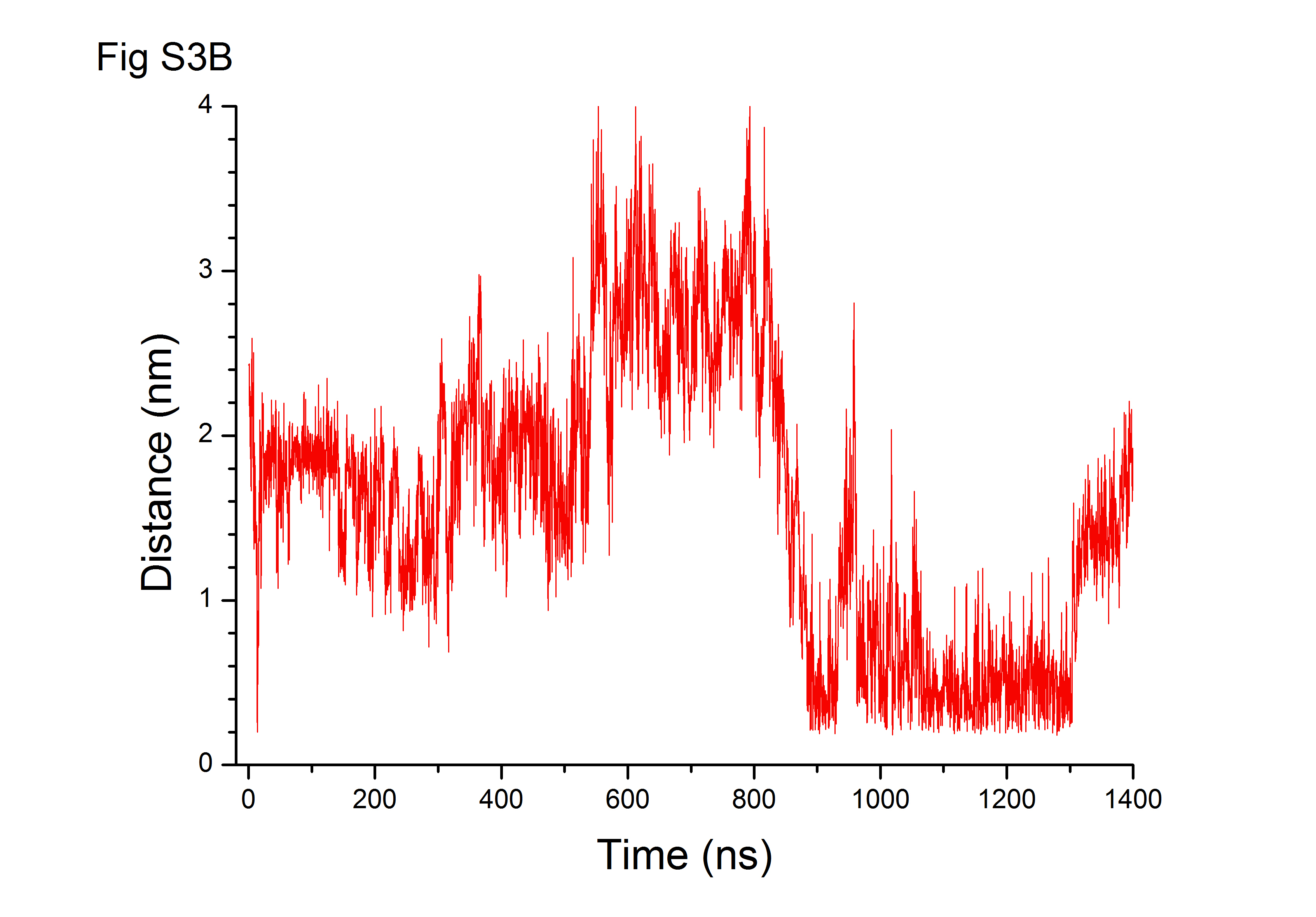


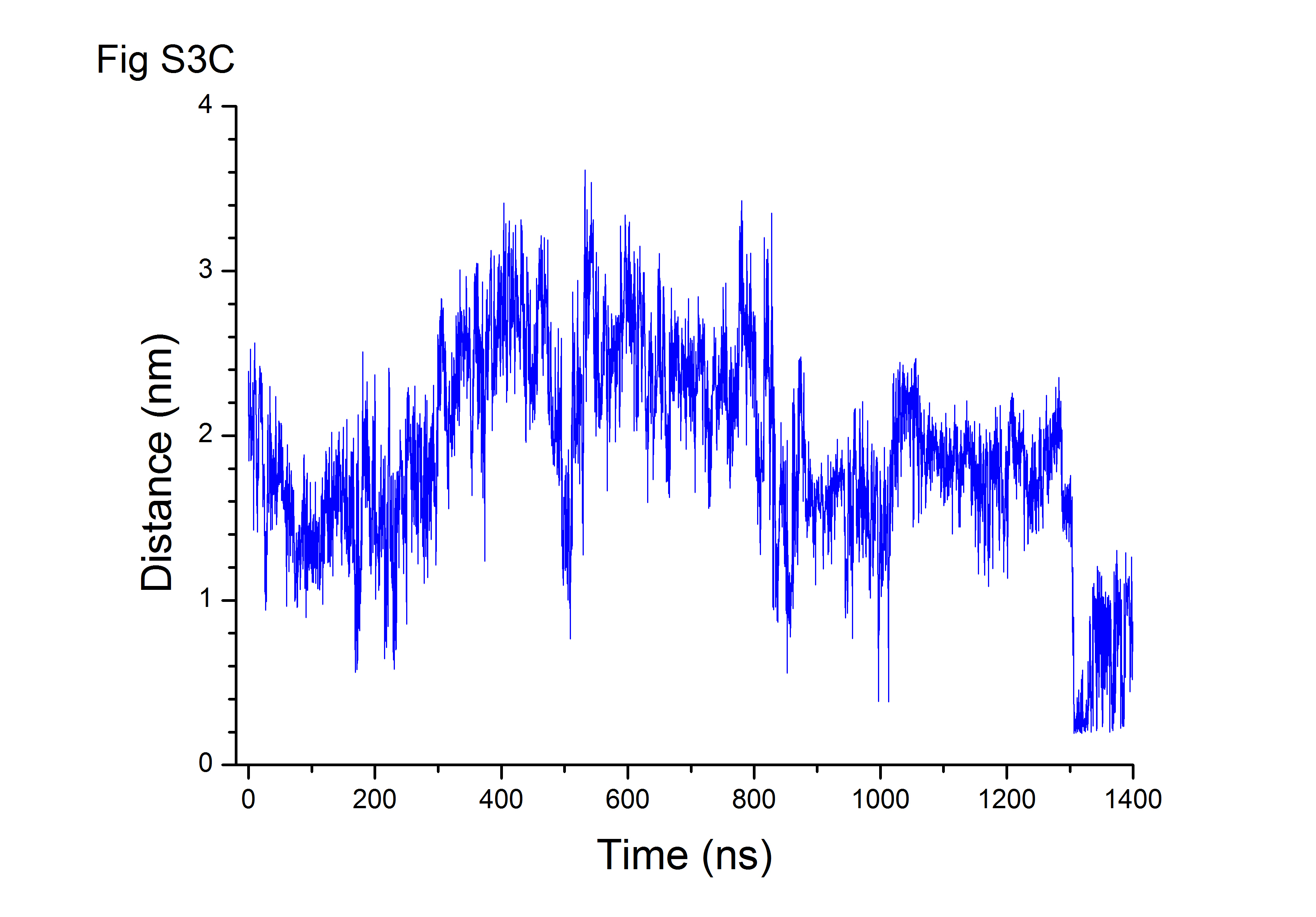


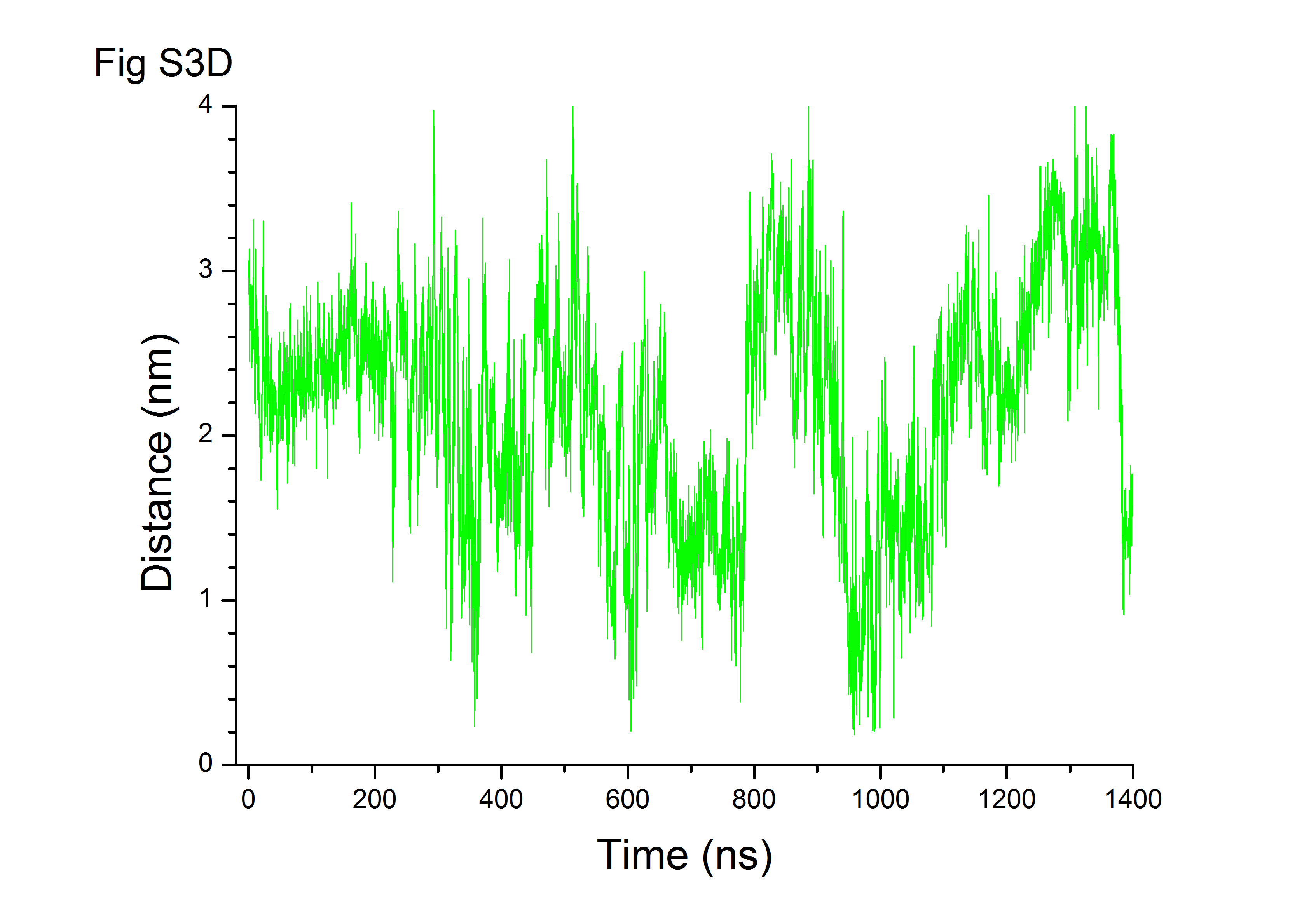


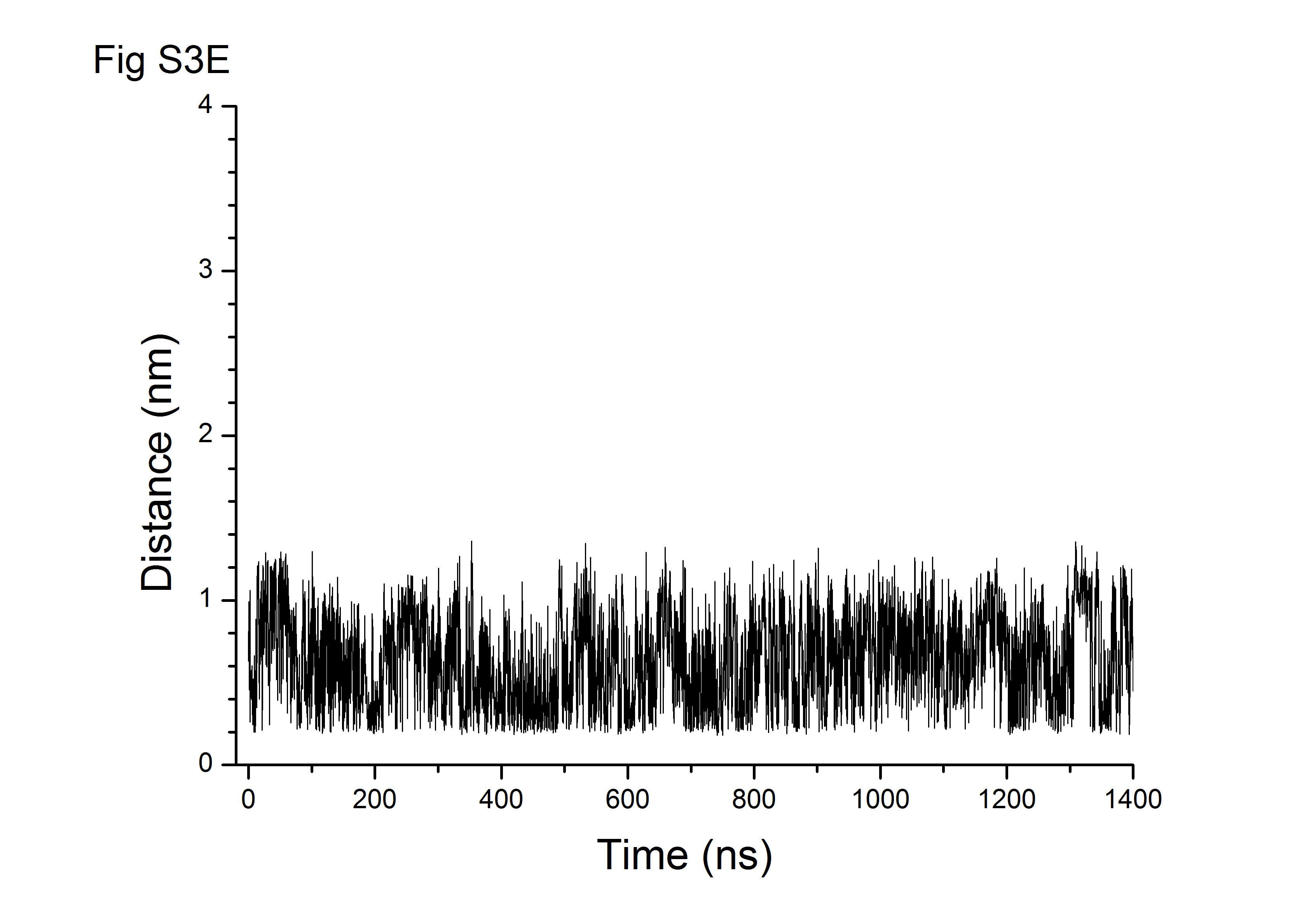

Supplement: Supplementary file 1 — Supplementary Information [file 41598_2018_25825_MOESM1_ESM.docx]
